# Utility of accessible SARS-CoV-2 specific immunoassays in vaccinated adults with a history of advanced HIV infection

**DOI:** 10.1038/s41598-024-58597-4

**Published:** 2024-04-09

**Authors:** Ludovica Ferrari, Alessandra Ruggiero, Chiara Stefani, Livia Benedetti, Lorenzo Piermatteo, Eleonora Andreassi, Federica Caldara, Drieda Zace, Matteo Pagliari, Francesca Ceccherini-Silberstein, Christopher Jones, Marco Iannetta, Anna Maria Geretti, The EVAN-COV Study Group, Lorenzo Ansaldo, Ada Bertoli, Francesco Bonfante, Neva Braccialarghe, Davide Checchi, Mirko Compagno, Giuseppe De Simone, Anna Maria Geretti, Sandro Grelli, Diletta Meloni, Tiziana Mulas, Lorenzo Piermatteo, Loredana Sarmati, Elisabetta Teti

**Affiliations:** 1 Department of Systems Medicine, University of Rome Tor Vergata, Rome, Italy; 2 Department of Infectious Diseases, Fondazione PTV, University of Rome Tor Vergata, Viale Oxford 81, 00133 Rome, Italy; 3 Department of Neurosciences, Biomedicine and Movement Sciences, School of Medicine, University of Verona, Verona, Italy; 4 Department of Biology, University of Rome Tor Vergata, Rome, Italy; 5 Department of Experimental Medicine, University of Rome Tor Vergata, Rome, Italy; 6 Laboratory of Experimental Animal Models, Division of Comparative Biomedical Sciences, Istituto Zooprofilattico Sperimentale Delle Venezie, Legnaro, Italy; 7 Department of Primary Care and Public Health, Brighton and Sussex Medical School, Falmer, UK; 8 Department of Infection, North Middlesex University Hospital, London, UK; 9 School of Immunity & Microbial Sciences, King’s College London, London, UK

**Keywords:** Immunology, Microbiology, Biomarkers, Diseases, Health care, Medical research

## Abstract

Accessible SARS-CoV-2-specific immunoassays may inform clinical management in people with HIV, particularly in case of persisting immunodysfunction. We prospectively studied their application in vaccine recipients with HIV, purposely including participants with a history of advanced HIV infection. Participants received one (n = 250), two (n = 249) or three (n = 42) doses of the BNT162b2 vaccine. Adverse events were documented through questionnaires. Sample collection occurred pre-vaccination and a median of 4 weeks post-second dose and 14 weeks post-third dose. Anti-spike and anti-nucleocapsid antibodies were measured with the Roche Elecsys chemiluminescence immunoassays. Neutralising activity was evaluated using the GenScript cPass surrogate virus neutralisation test, following validation against a Plaque Reduction Neutralization Test. T-cell reactivity was assessed with the Roche SARS-CoV-2 IFNγ release assay. Primary vaccination (2 doses) was well tolerated and elicited measurable anti-spike antibodies in 202/206 (98.0%) participants. Anti-spike titres varied widely, influenced by previous SARS-CoV-2 exposure, ethnicity, intravenous drug use, CD4 counts and HIV viremia as independent predictors. A third vaccine dose significantly boosted anti-spike and neutralising responses, reducing variability. Anti-spike titres > 15 U/mL correlated with neutralising activity in 136/144 paired samples (94.4%). Three participants with detectable anti-S antibodies did not develop cPass neutralising responses post-third dose, yet displayed SARS-CoV-2 specific IFNγ responses. SARS-CoV-2 vaccination is well-tolerated and immunogenic in adults with HIV, with responses improving post-third dose. Anti-spike antibodies serve as a reliable indicator of neutralising activity. Discordances between anti-spike and neutralising responses were accompanied by detectable IFN-γ responses, underlining the complexity of the immune response in this population.

## Introduction

Neutralising antibodies (NAbs) play a crucial role in the immune protection against SARS-CoV-2 by preventing the interaction between the spike (S) protein’s binding domain (RBD) and the human angiotensin-converting enzyme 2 (ACE2) receptor necessary for viral entry^1^. While serological assays are readily available to detect and quantify anti-spike (anti-S) antibody responses, they cannot distinguish between neutralising and total binding antibodies. The gold-standard approach for measuring neutralisation of SARS-CoV-2 is the Plaque Reduction Neutralization Test (PRNT), which employs live virus to test the ability of patient’s serum to neutralise infectivity and inhibit the formation of plaques in a cell monolayer^2^. However, the assay is time-consuming and labour-intensive and requires a high degree of technical expertise and Biosafety Level (BSL) 3 containment facilities^2^.

In response to these challenges, surrogate virus neutralization tests have emerged as a simpler, quicker and safer alternative^3,4^. Among these, the GenScript cPass assay received emergency use authorisation from the Food and Drug Administration (FDA) in November 2020^5^. The assay does not employ live virus or cells and can be completed within 1–2 h in a BSL2 laboratory. Previous studies have successfully used cPass to detect SARS-CoV-2 NAbs following a COVID-19 diagnosis^6^ or after vaccination of healthcare workers^6–8^ and other general populations^9–11^, as well as adolescents and young adults with childhood-onset rheumatic diseases^12^. The assay’s specificity was demonstrated with samples collected before the emergence of SARS-CoV-2^13–15^. Excellent performance was observed relative to neutralisation tests employing live virus^14,16–18^ or pseudo-typed virus^19^. A good correlation between surrogate virus neutralisation tests and serological tests measuring anti-S (RBD) antibodies has been described in general populations^20–22^. This raises doubts about the added value of surrogate virus neutralisation tests, but data within the setting of HIV infection are scarce.

Despite the benefits of effective antiretroviral therapy (ART), immunodysfunction can persist in people with HIV, especially among those who experienced marked immunological deterioration prior to the start of therapy^23,24^. Consequently, in the pre-vaccination era, a subset was found to have an increased risk of severe COVID-19 related outcomes^25–27^. In addition, a wide range of immune responses to SARS-CoV-2 vaccination have been described in this population^28^, suggesting that simplified neutralisation tests could be valuable. We conducted a prospective study in a cohort of adults with HIV who received the BNT162b2 vaccine in accordance with national guidelines. We deliberately included individuals with a history of advanced HIV infection, as potentially vulnerable to reduced vaccine immunogenicity, and measured SARS-CoV-2 antibody levels using anti-S (RBD) chemiluminescence immunoassay and cPass. To complement our approach, in participants lacking cPass neutralising responses post-third dose, we explored the use of the novel Roche SARS-CoV-2 IFN-γ release assay (IGRA)^29^. Finally, we assessed vaccine safety and tolerability and explored the association between the occurrence of severe systemic side effects and the strength of anti-S responses post-second vaccine dose.

## Methods

### Study population

The study enrolled adults with HIV who in March 2021 commenced SARS-CoV-2 vaccination in adherence to national guidelines. Recruitment was stratified into two groups of consecutive vaccine recipients living with HIV to ensure that approximately 50% of participants had a history of advanced HIV infection, defined as either a previous diagnosis of an AIDS-defining condition or a nadir CD4 count < 200 cells/mm^3^ without an AIDS-defining diagnosis. The primary vaccine series comprised two doses of the Pfizer/BNT162b2 vaccine administered with a 3-week interval; a subset of participants received a booster dose 6 months later. We obtained demographic and clinical information by reviewing medical records; plasma HIV-1 RNA levels and CD4 counts were retrieved from the most recent data available at the time of the first vaccine dose. A COVID-19 diagnosis was confirmed by testing respiratory samples for SARS-CoV-2 RNA or antigen. During the primary vaccine series, participants documented both solicited (i.e., specified) and unsolicited (i.e., spontaneously described) adverse events through structured questionnaires. Reporting of solicited side effects was in line with the methodology employed in the pivotal BNT162b2 vaccine trials^30,31^: participants were instructed to report any of the specified local (pain, redness and swelling at the injection site) and systemic (fever, chills, fatigue, headache, vomit, diarrhoea, muscle aches and joint aches) side effects occurring within the 7 days following each vaccine dose, and to indicate the grade (mild, moderate, severe) and duration (1–3 days; 4–5 days; > 5 days). Venous blood sampling for serum and plasma separation was scheduled for the time of the first vaccine dose (T0), 4 weeks after the second dose (T1) and 3 months after the third vaccine dose (T2). Serum and plasma were separated within 2 h of blood collection and stored at − 80°C. The study was approved by the Ethics Committee of Fondazione PTV, University of Rome Tor Vergata (reference: RS 40.21); all procedures were conducted according to relevant regulations and all participants provided written informed consent.

### Chemiluminescence assays for the detection of anti-Spike and anti-Nucleocapsid antibodies

Total antibodies to the spike protein (RBD) were quantified with the Elecsys® Anti-SARS-CoV-2 S assay on the Cobas e411 analyser (Roche Diagnostics, Rotkreuz, Switzerland)^32^. The assay reports results in U/mL, whereby 1 U/mL = 1 binding antibody unit (BAU)/mL^33^. Samples initially showing results above the upper limit of quantification (250 U/mL) were diluted in the assay diluent to obtain a quantified value. According to the manufacturer’s instructions, a cut-off of 0.8 U/mL indicates the presence of RBD-specific antibodies, whereas a cut-off of 15 U/mL is proposed to offer optimal correlation with the detection of neutralisation by PRNT (as determined with convalescent plasma from donors with SARS-CoV-2 infection)^32^. Total anti-nucleocapsid (anti-N) antibodies were detected qualitatively with the Elecsys® Anti-SARS-CoV-2 assay on the Cobas e411 analyser (Roche Diagnostics) according to the manufacturer’s instructions; a cut-off index (COI) ≥ 1.0 identified positive results.

### Plaque Reduction Neutralisation Test

The PRNT was performed as previously described^34^, using wild-type (Wuhan) SARS-CoV-2 and the Omicron BA.2 variant. Briefly, plasma samples were first heat-inactivated at 56 °C for 30 min. Two-fold dilutions were prepared in Dulbecco modified Eagle medium (DMEM) and mixed to a 1:1 ratio with a solution containing 20–25 foci forming units (FFUs) of SARS-CoV-2. After incubation for 1 h at 37 °C, 50 µL of the virus-plasma mixtures were added to confluent monolayers of Vero E6 cells in 96-wells plates and incubated for 1 h at 37 °C in 5% CO2. The inoculum was then removed and 100 μL of overlay solution (minimum essential medium [MEM], 2% foetal bovine serum [FBS], 100 U/mL penicillin, 100 U/mL streptomycin, 0.8% carboxy methyl cellulose) was added to each well. After incubation for 26 h, cells were fixed with a 4% paraformaldehyde (PFA) solution. Visualisation of plaques was obtained by immunocytochemical staining as described^34^. FFUs were counted after acquisition of pictures at a high resolution on a flatbed scanner. The neutralization titre was defined as the reciprocal of the highest dilution resulting in a reduction of the control plaque count > 50% (PRNT_50_). A titre of 1:10 was considered the seropositive threshold.

### cPass™ neutralisation test

The cPass SARS-CoV-2 Neutralization Antibody Detection Kit (GenScript, Piscataway, NJ, USA)^35^ was used to measure neutralisation of wild-type SARS-CoV-2 in a subset of participants at T0 and T1 and in all participants at T2, and neutralisation of the Omicron BA.2 variant in a subset of participants at T2. The method mimics the interaction between RBD and ACE2 in a blocking Enzyme-Linked Immunosorbent Assay (ELISA) format, whereby the presence of NAbs in the sample blocks binding of RBD to ACE2; the sample’s absorbance is inversely dependent on the NAb titre. The negative control must have an OD450 > 1.0, whereas the positive control must have an OD450 < 0.3. The seropositive threshold is > 30% neutralisation and results meeting this threshold are quantified in units (U)/mL using calibrators to prepare a standard curve. The assay was performed according to the manufacturer’s instructions^35^. Briefly, 1:10 diluted plasma samples, alongside negative and positive controls and calibrators, were first incubated with RBD protein labelled with horseradish peroxidase (HRP) at 37°C for 30 min; the mixture was then transferred to an ACE2-coated 96-well plate which was incubated at 37 °C for 15 min, followed by washing and read out. Two standard curves were established to quantify NAbs against wild-type SARS-CoV-2 (range 300–5 U/mL) and Omicron BA.2 (range 9600–38 U/mL). Plasma samples, standard curve calibrators, and positive and negative controls were tested in duplicate. The agreement between cPass and PRNT for neutralisation of wild-type SARS-CoV-2 and Omicron BA.2 was pre-determined using paired sample from 28 individuals with HIV who had received 3 vaccine doses. Neutralising activity against wild-type SARS-CoV-2 was detected in 27/28 samples by cPass and 28/28 samples by PRNT, yielding a qualitative concordance of 96.4%. Neutralising activity against Omicron BA.2 was detected in 25/28 and 28/28 samples respectively, yielding a qualitative concordance of 89.3%. Discordances were confirmed by repeat testing.

### IFN-γ release assay

The Roche Diagnostics Elecsys® IGRA SARS-CoV-2 test was used on the Cobas e411 analyser to measure IFN-γ release in response to stimulation with SARS-CoV-2 antigens, as described by the manufacturer^29^. Briefly, whole blood was collected in 3 cobas® IGRA SARS-CoV-2 tubes, comprising the antigen tube coated with 189 different SARS-CoV-2 peptides representing the viral membrane, nucleocapsid, spike, and non-structural proteins; the positive mitogen control tube; and the negative control tube. After incubation for 16–24 h at 37 °C, plasma was separated by centrifugation at 2000 RCF for 5 min and stored at 80 °C for ≤ 4 weeks prior to measuring IFN-*γ* release in an automated electrochemiluminescence immunoassay. Readouts were standardised against the first British interferon gamma (human leukocyte-derived) standard (NIBSC code 82/587) and reported in IU/mL. Valid read-outs required a positive control value ≥ 1.0 IU/mL and a negative control value ≤ 0.3 IU/mL. The test tube readout was obtained after subtracting the negative control readout.

### Statistical analysis

The characteristics of the study population were summarised as categorical variables (expressed as counts and proportions) and continuous variables (expressed as medians with interquartile range [IQR]). The category male to female trans was analysed together with the category assigned male at birth. The occurrence of solicited and unsolicited side effects was described as proportions documenting side effects in the questionnaires. The Chi-square test was used to compare proportions with solicited side effects in the study population versus the pivotal BNT162b2 trials^30^. Proportions who took pain relief medication after the first vs. the second vaccine dose were compared by the McNemar’s test. Factors associated with the occurrence of severe side effects after the second vaccine dose were explored by logistic regression analysis. Factors associated with anti-S titres following the second vaccine dose were explored by linear regression analysis following log transformation of anti-S titres; coefficients were converted into fold-changes. Due to co-linearity, the models adjusted separately for ethnicity or transmission group and included the CD4 count in place of the CD4:CD8 ratio. The models also adjusted for age and SARS-CoV-2 exposure prior to starting vaccination; the latter was defined as a previous COVID-19 diagnosis and/or detection of anti-S and/or anti-N antibodies at T0. Anti-S titres (in log_10_ U/mL) at T1 were compared between participants that did and did not enter the extension cohort using the Mann–Whitney U test. Anti-S and NAb titres (in log_10_ U/mL) by time point (T0, T1, T2) and neutralisation of wild-type SARS-CoV-2 vs. Omicron BA.2 at T2 were compared with the Wilcoxon signed-rank test. The correlation between anti-S and NAb titres (in log_10_ U/mL) was analysed using Spearman’s rank. Anti-S and NAb titres (in log_10_ U/mL) by CD4 count stratum (above or below 350 cells/mm^3^) were compared with the Mann–Whitney U test. The analyses were performed using STATA (Version 18.0 College Station, Texas, USA) and GraphPad Prism (Version 10.1.0, San Diego, CA, USA).

## Results

### Characteristics of the population at study entry

The study enrolled 250 consecutive adults with HIV who received their first BNT162b2 vaccine dose between March and September 2021 (Table 1). Of these, 249/250 (99.6%) received the second vaccine dose within a median of 3 weeks of the first (IQR 3, 3). The median age of the participants was 48 years, ranging from 22 to 78. There was a predominance of white men but also diversity in terms of region of origin, ethnicity, HIV transmission group, self-reported socio-economic status and HIV history. Nearly all participants, except for one elite controller, were receiving ART, primarily consisting of tenofovir alafenamide plus emtricitabine (168/249, 67.5%) and an integrase inhibitor (179/249, 71.9%). Most participants (226/250, 90.4%) showed plasma HIV-1 RNA suppression (< 50 copies/mL) and well-maintained CD4 counts (median 710 cells/mm^3^), as measured a median of 2 weeks (IQR 0, 10) before the first vaccine dose. However, 24/250 (9.6%) had viremia, typically at low level (median 156 HIV-1 RNA copies/mL [IQR 97, 1162]). Additionally, 37/250 (14.8%) had a CD4 count < 350 cells/mm^3^ and the median CD4/CD8 ratio was only 0.8. Reflecting the recruitment strategy, nearly half (118/250; 47.2%) had experienced advanced HIV infection, either a previous AIDS-defining diagnosis (74/250, 29.6%) or a nadir CD4 count < 200 cells/mm^3^ (44/250, 17.6%). Approximately one third (89/250, 35.6%) had ≥ 1 recorded comorbidity, with hypertension and cardiovascular disease being the most commonly documented comorbidities. A total of 45/250 (18.0%) participants had data indicating proven or likely pre-vaccination exposure to SARS-CoV-2. This included 21/250 (8.4%) that had previously received a diagnosis of COVID-19, with a median of 7 months (IQR 5, 9) elapsing prior to the first vaccine dose and 24/201 (11.9%) without a COVID-19 diagnosis but with anti-N and/or anti-S antibodies before the start of vaccination. Suggesting previous exposure, 40/201 (19.9%) participants had anti-S antibodies at T0 and a similar proportion had anti-N antibodies (Table 1), with 37/201 (18.4%) showing both antibodies.

**Table 1:** Baseline characteristics of the study population.

Characteristics	Primary cohort		Extension cohort^a^	
	N = 250		N = 42	
Age, median years (IQR)	48	41–56	55	50–62
Sex, n (%)				
Female at birth	64	25.6	11	26.2
Male at birth	186^b^	74.4	31	73.8
Country or region of birth, n (%)				
Italy	187	74.8	37	88.1
Sub-Saharan Africa	26	10.4	3	7.1
Latin America	16	6.4	1	2.4
Eastern Europe	12	4.8	1	2.4
Other	9	3.6	0	0
Ethnicity, n (%)				
White	200	80.0	38	90.5
Black African	26	10.4	3	7.1
Hispanic/Latino	16	6.4	1	2.4
Other	8	3.2	0	0
Transmission group, n (%)				
Heterosexual	92	36.8	19	45.2
MSM	109	43.6	15	35.7
Trans MTF	8	3.2	0	0
IDU	40	16.0	8	19.0
Vertical	1	0.4	0	0
Socio-economic status, n (%)				
Full/Part-time work	133	53.2	18	42.9
Unemployed	50	20.0	6	14.3
Retired/Home keeper	51	20.4	14	33.3
Other/Prefer not to say	16	6.4	4	9.5
Duration HIV diagnosis, median years (IQR)	8	4–14	5	2–16
History of advanced HIV infection^c^, n (%)	118	47.2	30	71.4
Nadir CD4 count, median cells/mm^3^ (IQR)	225	64–414	93	39–217
CD4 count, median cells/mm^3^ (IQR)	706	470–942	433	233–665
CD4 cells/mm^3^, n (%)				
> 500	179	71.6	13	31.0
350–500	34	13.6	13	31.0
200–349	20	8.0	8	19.0
< 200	17	6.8	8	19.0
CD4:CD8 ratio, median (IQR)	0.8	0.5–1.2	0.6	0.3–1.0
ART duration, median years (IQR)	7	4–11	5	2–14
ART regimen class, n (%)				
INSTI	179	71.6	31	73.8
NNRTI	27	10.8	2	4.8
PI/b	27	10.8	4	9.5
Other	16	6.4	5	11.9
None	1	0.4	0	0
HIV-1 RNA ≥ 50 copies/mL, n (%)	24	9.6	11	26.2
Recorded comorbidities, n (%)				
0	161	64.4	24	57.1
1	51	20.4	11	26.2
2	26	10.4	5	11.9
≥ 3	12	4.8	2	4.8
Obesity^d^	16	6.4	4	9.5
Diabetes	17	6.8	1	2.4
CVD	29	11.6	5	11.9
Hypertension	62	24.8	15	35.7
CKD	11	4.4	2	4.8
COPD	4	1.6	1	2.4
Cancer	5	2.0	0	0
Dementia	2	0.8	0	0
Previous COVID-19 diagnosis, n (%)	21^e^	8.4	7f	16.7
Anti-N, n (%)				
Positive	40^g^	16.0	8^h^	19.0
Negative	204	81.6	34^i^	81.0
Not available	6	2.4	0	0
Anti-S, n (%)				
Positive	40	16.0	9	21.4
Negative	161	64.4	29	69.0
Not available	49	19.6	4	9.5
Anti-S titre, median U/mL (IQR)^j^	47	18–208	22	13–45

^a^The extension cohort comprised participants who received a third vaccine dose after completion of the primary vaccine series.

^b^Included 8 male to female trans participants.

^c^Either a previous AIDS-defining diagnosis or a nadir CD4 count < 200 cells/mm^3^.

^d^Body mass index > 30 kg/m^2^.

^e^17/21 and 19/21 had anti-N antibodies and anti-S antibodies at T0, respectively; in the 4 participants lacking anti-N antibodies, the COVID-19 diagnosis was made 1, 7, 7, and 12 months before anti-N testing and 2/4 had anti-S antibodies at T0.

^f^All had anti-N antibodies and anti-S antibodies at T0.

^g^37/40 with anti-N antibodies also had anti-S antibodies at T0.

^h^All also had anti-S antibodies at T0.

^i^7/34 acquired anti-N antibodies between T1 and T2.

^J^Among participants with detectable anti-S at T0.

IQR, Interquartile range; MTF, Male to female; MSM, Men who have sex with men; IDU, injecting drug use; ART, Antiretroviral therapy; INSTI, Integrase strand transfer inhibitor; NNRTI, Non-nucleoside reverse transcriptase inhibitor; PI/b, Boosted protease inhibitor; CVD, Cardiovascular disease; CKD, Chronic kidney disease; COPD, Chronic Obstructive Pulmonary Disease; Anti-N, Anti-Nucleocapsid antibodies; Anti-S, Anti-Spike antibodies; T0, Time zero (pre-vaccination time point).

### Side effects of primary vaccination

**Figure 1:**
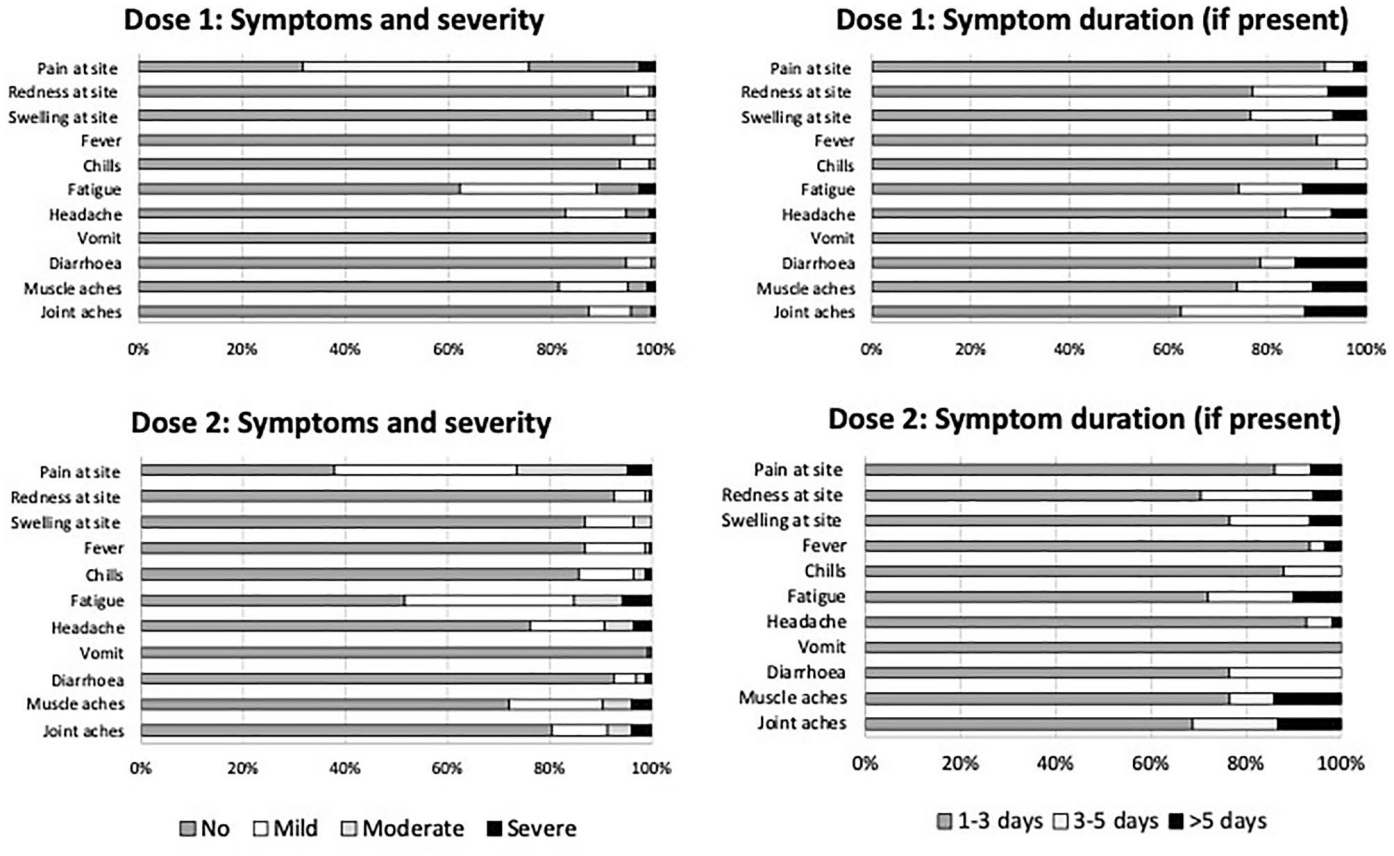
Solicited side effects, with severity and duration, in the 7 days after each of two BNT162b2 vaccine doses (n = 245 post-first dose and n = 230 for post-second dose).

The structured questionnaires documenting solicited and unsolicited side effects were returned by 245/250 (98.0%) participants post-first vaccine dose and by 230/249 (92.4%) post-second dose. The individual who declined the second vaccine dose reported a superficial thrombophlebitis 7 days after the first dose, which was not considered related to vaccination. Proportions documenting solicited side effects mirrored observations from the pivotal BNT162b2 trials^30^ (Supplementary Fig. 1). Overall, 191/245 (78.0%) participants documented solicited events post-first dose, including local side effects in 69 (28.2%), systemic side effects in 19 (7.8%), and a combination of both in 103 (42.0%). Post-second dose, 175/230 (76.1%) documented solicited events, including local side effects in 37 (16.1%), systemic side effects in 30 (13.0%) and a combination of both in 108 (47.0%). Pain at the injection site, fatigue, muscle and joint aches and headache were the main solicited side effects documented (Supplementary Table 1), and most were mild and lasted for < 3 days (Fig. 1). A total of 18/245 (7.3%) and 21/230 (9.1%) participants documented unsolicited side effects post-first and post-second dose, respectively; the most common were light-headiness, nausea and loss of appetite (Supplementary Table 1). There were 20/245 (8.2%) and 32/230 (13.9%) participants reporting severe side effects post-first dose and post-second dose, respectively, with 6/245 (2.4%) and 13/230 (5.7%) describing ≥ 2 severe side effects. The use of pain-relief medication was reported by 33/245 (13.5%) participants post-first dose and 56/230 (24.3%) post-second dose (odds ratio 2.79; 95% confidence interval [CI] 1.48, 5.55; p = 0.001), whereas only a few sought medical care in relation to side effects (5/245, 2.0% vs. 5/230, 2.2%). No associations were observed between the reporting of severe side effects and available demographic or clinical characteristics (not shown), although severe systemic side effects post-second dose were marginally more common among women (11/57 with questionnaire data, 19.3%) compared with men (15/173, 8.7%).

### Anti-S antibody responses to the primary vaccine series

**Figure 2:**
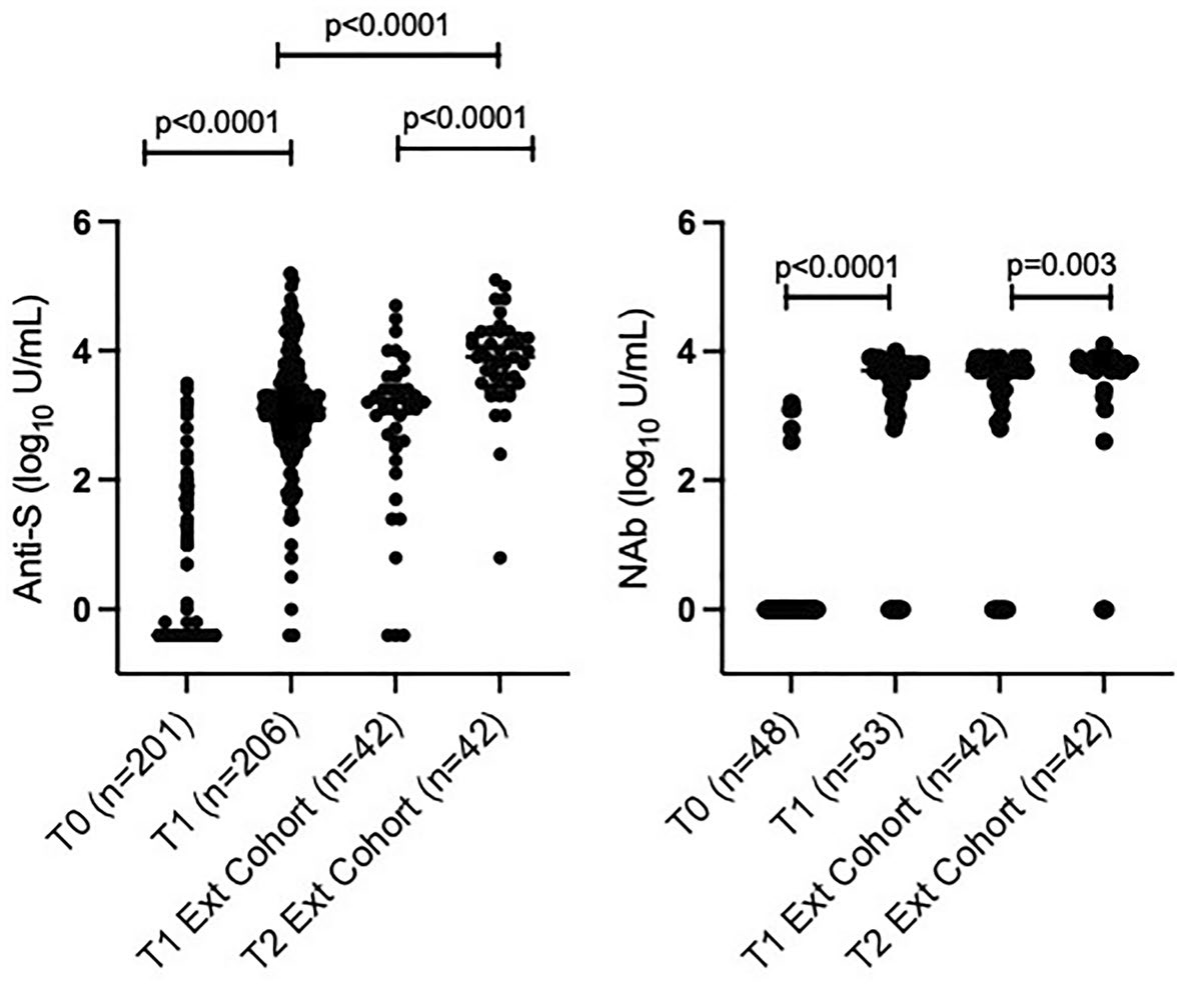
Anti-spike (S) (left) and neutralising antibody (NAb) (right) titres at T0, T1 and T2. Neutralising activity was measured by cPass against wild-type SARS-CoV-2.

Among the 249 participants who received 2 vaccine doses, 206 (82.7%) underwent sampling a median of 4 weeks (IQR 3, 5) post-second dose (T1) and 202/206 (98.0%) had quantifiable anti-S antibodies with median titres of 3.1 log_10_ U/ml (IQR 2.8, 3.4) (Fig. 2). In the univariable analysis (Table 2), factors associated with higher anti-S antibody titres included previous SARS-CoV-2 exposure, African ethnicity and higher CD4:CD8 ratio. Factors associated with lower titres included a history of IDU, CD4 counts < 350 cells/mm^3^ and HIV-1 RNA levels ≥ 50 copies/mL. There was also a trend for higher anti-S titres among participants who documented severe systemic side effects post-second dose. After adjustment, factors independently associated with T1 anti-S titres included prior SARS-CoV-2 exposure, African ethnicity, history of IDU, CD4 counts < 350 cells/mm^3^ and HIV-1 RNA levels ≥ 50 copies/mL.

**Table 2:** Factors associated with anti-S antibody titres after two doses of the BNT162b2 vaccine (n = 206).

Variable	Univariate				Multivariable Model 1				Multivariable Model 2		
	FC	95% CI	P-value	FC		95% CI	P-value	FC		95% CI	P-value
Age											
Per 5 years older	0.93	0.83–1.99	0.35	1.01		0.91–2.51	0.88	0.99		0.91–2.34	0.91
Sex at birth											
Female (n = 53) vs. Male (n = 153)	0.83	0.93–1.58	0.57								
Ethnicity											
White (n = 172)	REF							REF			
Black-African (n = 21)	12.01	5.60–28.18	< 0.01					3.09		1.51–6.16	< 0.01
Hispanic/Latino (n = 7)	0.15	0.02–1.01	0.05					0.37		0.08–1.51	0.16
Transmission group											
Heterosexual (n = 75)	REF			REF							
MSM (n = 92)	0.67	0.36–1.26	0.15	0.79		0.49–1.23	0.29				
IDU (n = 35)	0.27	0.12–0.63	< 0.01	0.43		0.24–0.79	< 0.01				
Duration HIV diagnosis											
Per 5 years longer	0.95	0.81–1.12	0.58								
History advanced HIV infection^a^											
Yes (n = 107) vs. no (n = 99)	0.79	0.45–1.38	0.42								
CD4 count, cells/mm^3^											
> 500 (n = 147)	REF			REF				REF			
350—500 (n = 28)	1.31	0.61–2.81	0.50	1.20		0.68–2.14	0.54	1.12		0.63–2.04	0.69
200—349 (n = 17)	0.22	0.08–0.59	< 0.01	0.32		0.15–0.67	< 0.01	0.38		0.17–0.81	0.01
< 200 (n = 14)	0.04	0.01–0.13	< 0.01	0.06		0.02 -0.15	< 0.01	0.08		0.04–0.19	< 0.01
CD4:CD8 ratio^b^											
Per 0.1 unit higher	2.08	1.20–3.54	< 0.01								
ART duration											
Per 5 years longer	1.01	0.98–1.22	0.93								
HIV-1 RNA ≥ 50 copies/mL											
Yes (n = 22) vs. No (n = 184)	0.12	0.04–0.31	< 0.01	0.47		0.23–0.96	0.04	0.43		0.21 -0.91	0.02
Recorded comorbidities											
0 (n = 127)	REF										
1 (n = 44)	0.79	0.38–1.54	0.49								
≥ 2 (n = 35)	1.58	0.74–3.54	0.23								
Previous COVID-19 exposure^b^											
Yes (n = 35) vs. No (n = 171)	27.54	15.13–50.12	< 0.01	19.90		15.50–34.67	< 0.01	15.48		8.71–27.54	< 0.01
Severe systemic side effects^c^											
Yes (n = 20) vs. No (n = 181)	2.39	0.93–6.16	0.07								

Participants received two doses of the BNT162b2 vaccine administered with a 3-week interval and anti-S titres were measured a median of 4 weeks (IQR 3, 5) after the second vaccine dose.

FC = Fold-change; CI = Confidence interval; MSM, Men who have sex with men; IDU, injecting drug use; ART, Antiretroviral therapy; INSTI, Integrase strand transfer inhibitor; NNRTI, Non-nucleoside reverse transcriptase inhibitor; PI/b, Boosted protease inhibitor; CVD, Cardiovascular disease; CKD, Chronic kidney disease; COPD, Chronic Obstructive Pulmonary Disease; Anti-N, Anti-Nucleocapsid antibodies; Anti-S, Anti-Spike antibodies; T0, Time zero (pre-vaccination time point).

^a^Defined as either a previous AIDS-defining diagnosis or nadir CD4 count < 200 cells/mm^3^.

^b^Defined as a COVID-19 diagnosis and/or detection of anti-S and/or anti-N antibodies pre-vaccination.

^c^Documented after the second vaccine dose.

### Anti-S and neutralising antibody responses after the third vaccine dose in the extension cohort

Between September 2021 and April 2022, a median of 6 months (IQR 5, 7) post-second dose, 42 participants received a third vaccine dose. Their characteristics are shown in Table 1. At T1, median anti-S titres were 3.2 log_10_ U/mL (IQR 2.6, 3.4) (Fig. 2) and similar to those of the population that did not enter the extension cohort [median 3.1 log_10_ U/ml (IQR 2.8, 3.5). A median of 14 weeks (IQR 10, 19) post-third dose (T2), anti-S titres increased to a median of 3.9 log_10_ U/mL (IQR 3.5, 4.2) (Fig. 2). Neutralising activity against wild-type SARS-CoV-2 also increased significantly, from a median of 96% (IQR 70, 98) at T1 to a median of 98% (IQR 97, 98) at T2, with median NAb titres of 3.7 log_10_ U/mL (IQR 3.1, 3.8) and 3.8 log_10_ U/mL (IQR 3.7, 3.8), respectively (Fig. 2). At T2, neutralisation activity against Omicron BA.2 was reduced compared with wild-type SARS-CoV-2 [median 83% (IQR 65, 92) vs. 98% (IQR 97, 98); p < 0.001] (Supplementary Fig. 2). As a potential contributor to enhanced immune responses, exposure to SARS-CoV-2 between T1 and T2 was documented in 9/42 (21.4%) participants, based on a COVID-19 diagnosis (n = 4) and/or seroconversion for anti-N antibodies. At T1, median antibody titres in participants with vs. those without subsequent SARS-CoV-2 exposure were 2.6 (IQR 1.6, 3.3) vs. 3.1 (IQR 2.4, 3.3) log_10_ U/mL for anti-S antibodies and 3.4 (IQR 1.4, 3.7) vs. 3.7 (IQR 3.2, 3.7) log_10_ U/mL for NAbs.

### Correlation between anti-S and neutralising antibody titres

**Figure 3:**
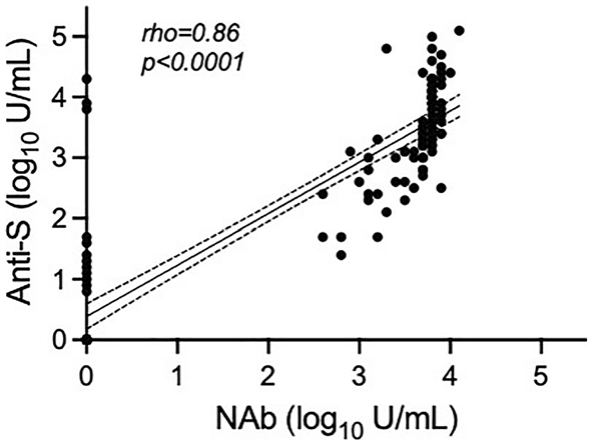
Correlation between anti-Spike (S) and neutralising antibody (NAb) titres measured at T0, T1 and T2 (n = 144). Neutralising activity was measured by cPass against wild-type SARS-CoV-2.

Detection of anti-S antibodies coincided with the detection of neutralising activity against wild-type SARS-CoV-2 by cPass in 5/10 (50%) samples at T0, 45/49 (91.8%) samples at T1, and 40/43 (93.0%) samples at T2 (Table 3). Anti-S titres were median 3.4 log_10_/mL (IQR 3.0, 4.0) and 1.4 log_10_ U/ml (IQR 1.1, 3.3) in samples with and without neutralising activity, respectively. Anti-S and NAb titres showed a large positive correlation both when pooling all the paired results (n = 144; rho 0.86; p < 0.0001) (Fig. 3) and when limiting the correlation analysis to T1 and T2 samples (n = 96; rho 0.67; p < 0.0001). Across time points, detection of anti-S antibodies in the absence of cPass neutralising activity occurred in 12/144 (8.3%) samples; 4/12 discordances were resolved by applying the manufacturer’s suggested anti-S titre threshold of 15 U/mL^33^ and a further 5/12 had anti-S titres between 18 and 55 U/mL, leaving 3/144 (2.1%) samples with anti-S titres > 3.8 log_10_ U/mL but undetectable NAbs by cPass (Table 3).

**Table 3:** Neutralising activity against wild-type SARS-CoV-2 in relation to anti-S antibody status (n = 144).

Anti-S	T0 NAb			T1 NAb			T2 NAb		
	Positive	Negative	Total	Positive	Negative	Total	Positive	Negative	Total
Positive	5	5^a^	10	45	4^b^	49	40	3^c^	43
Negative	0	38	38	0	4	4	0	0	0
Total	5	43	48	45	8	53	40	3	43

Neutralising activity was measured by cPass.

^a^Anti-S titres: 9, 13, 18, 22, 37 U/mL.

^b^Anti-S titres: 7, 23, 55 and 7227 U/mL.

^c^Anti-S titres: 7, 7012 and 19,210 U/mL.

### Anti-S and neutralising antibody responses in relation to CD4 cell counts

**Figure 4:**
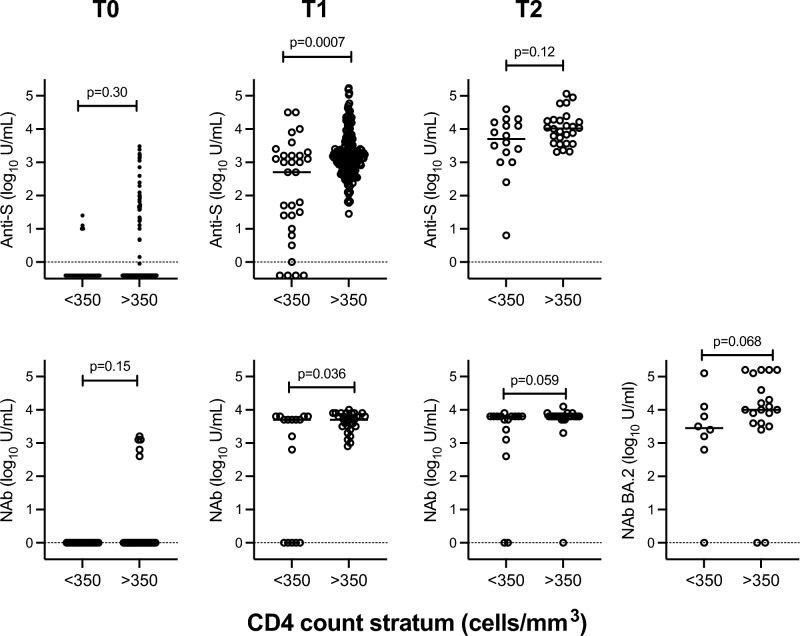
Top panel: Anti-spike (S) titres by CD4 count stratum at T0 (n = 28 vs. n = 173), T1 (n = 31 vs. n = 175) and T2 (n = 16 vs. n = 26). Bottom panel: Neutralising antibody (NAb) titres by CD4 count stratum measured by cPass against wild-type SARS-CoV-2 at T0 (n = 16 vs. n = 32), T1 (n = 16 vs. n = 31) and T2 (n = 16 vs. n = 26) and also against Omicron BA.2 at T2 (n = 8 vs. n = 20).

At T1, anti-S titres were higher at CD4 counts > 350 cells/mm^3^ [median 3.2 log_10_ U/mL (IQR 2.8, 3.5); n = 175] compared with lower CD4 counts [median 2.7 log_10_ U/mL (IQR 1.0, 3.2); n = 31) (p = 0.0007) (Fig. 4). NAb titres, measured by cPass in a subset of participants at T1, were similarly higher at CD4 counts > 350 cells/mm^3^ [median 3.7 log_10_ U/mL (IQR 3.5, 3.9); n = 31] compared with lower CD4 counts [median 3.7 log_10_ U/mL (IQR 0, 3.8); n = 16) (p = 0.036). At T2, median anti-S titres were 4.0 log_10_ U/mL (IQR 3.6, 4.3) at CD4 counts > 350 cells/mm^3^ (n = 26) vs. 3.7 log_10_ U/mL (IQR 3.1, 4.2) at lower CD4 counts (n = 16) (p = 0.12), whereas median NAb titres were 3.8 log_10_ U/mL (IQR 3.8, 3.8; n = 26) vs. 3.8 log_10_ U/mL (IQR 3.2, 3.8; n = 16), respectively (p = 0.059). The characteristics of participants with reduced antibody responses to vaccination are detailed in Table 4. Three patterns were observed. The first was lack of anti-S and NAbs at T1 but development of both at T2 (n = 2/42, 4.8%; ID 67, 149). The second was detection of anti-S at T1 and T2, but lack of NAbs until T2 (n = 3/42, 7.1%; ID 62, 66, 97). The third was detection of anti-S at T1 and T2, but lack of NAbs at both time points (n = 3/42, 7.1%; ID 01, 79, 173). The 3 participants lacking NAb at T2 by cPass had detectable SARS-CoV-2 specific IFNγ responses by IGRA, with IFN-*γ* levels of 1.2, 0.1, and 0.2 IU/mL, respectively.

**Table 4:** Individual participants with reduced antibody responses to vaccination.

ID	Characteristics at study entry (T0)							SARS-CoV-2 exposure^a^		Interval 2nd vaccine dose—T1	Interval 3rd vaccine dose—T2	Anti-S log_10_ U/mL			Neutralisation % (NAb log_10_ U/mL)		
	Age, sex Ethnicity, Group	Nadir CD4 cells/mm^3^	CD4 cells/mm^3^	CD4:CD8 ratio	HIV-1 RNA copies/mL	Years of ART	Co-morbidity	Pre-vaccine	Post-vaccine			T0	T1	T2	T0	T1	T2
67	53, F, W, IDU	120	235	0.6	50	30	Obesity	No	No	5 weeks	8 weeks	ND	Neg	4.1	ND	Neg	98% (3.8)
149	57, F, L, Heter	17	112	0.1	< 50	1	None	No	Yes	11 weeks	23 weeks	Neg	Neg	4.3	Neg	Neg	99% (3.8)
275^b^	42, M, W, Heter	106	311	0.4	< 50	10	None	No	Yes	3 weeks	NA	ND	Neg	2.5	ND	Neg	98% (3.9)
62	57, M, W, Heter	14	175	0.2	69	1	None	No	No	5 weeks	20 weeks	Neg	1.4	3.0	Neg	Neg	63% (3.1)
66	52, M, W, MSM	166	260	0.4	464	0.5	HT	No	Yes	5 weeks	17 weeks	Neg	1.7	4.2	Neg	Neg	97% (3.9)
97	55, M, W, IDU	103	140	0.5	< 50	23	None	No	No	3 weeks	16 weeks	Neg	0.9	2.4	Neg	Neg	33% (2.6)
01	68, M, W, MSM	536	966	1.0	< 50	8	None	No	No	3 weeks	13 weeks	Neg	3.1	4.3	Neg	Neg	Neg^c^
79	53, M, W, IDU	83	158	0.1	< 50	1	HT	No	No	4 weeks	19 weeks	ND	Neg	0.8	ND	Neg	Neg
173	65, M, W, Heter	6	89	0.1	< 50	1	None	Yes	No	4 weeks	19 weeks	1.1	3.9	3.8	Neg	Neg	Neg^c^

Neutralising capability was measured by cPass.

T0, Time-point zero (pre-vaccination); T1, Sampling time-point after the second vaccine dose; T2, Sampling time-point after the second vaccine dose; Anti-S, Anti-Spike antibodies; NAb, Neutralising antibodies (against wild-type SARS-CoV-2); F, Female at birth; M, Male at birth; W, White; L, Latino; IDU, Injecting drug use; Heter, Heterosexual; MSM, Men who have sex with men; ART, Antiretroviral therapy; HT, Hypertension; ND, Not done; Neg, Negative (undetectable).

^a^Defined as a COVID-19 diagnosis and/or detection of anti-N and/or anti-S antibodies pre vaccination, and as a COVID-19 diagnosis and/or anti-N seroconversion between T1 and T2.

^b^Participant ID 275 did not receive a third vaccine dose (and was not included in the extension cohort), but had a COVID-19 diagnosis and received SARS-CoV-2 monoclonal antibodies (Ronapreve) in December 2021 with the T2 sample collected 11 weeks later.

^c^Also negative for neutralisation activity against Omicron BA.2.

## Discussion

In 2021–2022, Italy adopted strict policies on vaccination against COVID-19, making it mandatory for large sectors of the population to receive primary vaccination followed by a booster dose. In the age range 40–59, 88% received primary vaccination and 74% received the booster dose as of 24/09/2023^36^. We studied responses to the BNT162b2 vaccine in a prospective cohort of adults living with HIV, deliberately including a large proportion of individuals with a history of advanced HIV infection. At the time of vaccination, most participants had well-suppressed HIV-1 RNA and well-maintained CD4 counts. However, reflecting the spectrum of HIV disease within the cohort, a subset had detectable viremia and low CD4 counts.

With the exception of one individual, all participants completed primary vaccination with two vaccine doses; a smaller subset also received a third dose. Primary vaccination was well tolerated. Most individuals reported mild and short-lived solicited side effects, typically pain at the injection site, fatigue, muscle and joint aches and headache, whereas the most common unsolicited side effects were light-headiness, nausea and loss of appetite. Systemic side effects were more often reported after the second vaccine dose compared with the first dose, but there was no evidence of an increased risk of adverse events or of a different pattern of side effects relative to general trial populations^30,31^. There was also no association between HIV-related parameters and the likelihood of reporting severe side effects. Interestingly, post-second dose anti-S titres were higher in individuals reporting severe systemic side effects, but the wide confidence interval precludes definitive conclusions regarding the association.

Following primary vaccination, the large majority of participants demonstrated quantifiable anti-S antibodies. Titres differed widely, being predictably higher among participants with prior SARS-CoV-2 exposure, but also among those of African ethnicity. The association between higher anti-S titres and African ethnicity was independent of other variables, including a prior SARS-CoV-2 exposure. Previous studies similarly reported that African ethnicity was associated with enhanced humoral responses to vaccination against pathogens such as influenza, rubella, measles and pertussis, relating the finding to distinct pre-vaccination gene expression profiles^37^. Consistent with these observations, in a large trial, the clinical efficacy of the BNT162b2 vaccine was 95.2% among Caucasians (n = 31,266) and 100% in Afro-Caribbean or African Americans (n = 3492)^31^.

A history IDU was independently associated with reduced anti-S titres after primary vaccination. Decreased antibody responses to vaccination against agents such as tetanus, influenza, or hepatitis A and B have been previously reported in the context of IDU, although there is no clear evidence of decreased clinical protection from these infections^38^. However, a recent study indicated that the effectiveness of two COVID-19 vaccine doses against testing positive for SARS-CoV-2 infection was markedly lower among people with HIV who had a history of IDU compared with those without such history^39^. These findings need confirmation in studies that differentiate between current and past IDU.

Anti-S titres after primary vaccination were also lower among participants with CD4 counts < 350 cells/mm^3^ or detectable HIV-1 RNA levels. Low CD4 counts and viremia are known to reduce vaccine immunogenicity in people living with HIV^40^ and were previously found to reduce responses to SARS-CoV-2 mRNA vaccines^41–44^. Notably, we demonstrated the detrimental effects of viremia at low HIV-1 RNA levels, and independently of CD4 counts. The administration of a third vaccine dose significantly enhanced anti-S and neutralising responses, reducing the difference between people with CD4 counts < 350 cells/mm^3^ and those with higher CD4 counts. Thus, optimising HIV suppression^45^ and adopting appropriate vaccination and boosting strategies^44,46,47^ are important elements of effective prevention in this population. The finding that a third vaccine dose can “rescue” antibody responses in people with low CD4 cell counts is in agreement with previous reports^46–48^. However, in line with our data, there is published evidence that some individuals with CD4 counts < 350 cells/mm^3^ continue to experience a response gap after three vaccine doses^43^. The variable findings may reflect differences in the composition of study populations and any boosting effect of SARS-CoV-2 infection^49^. In our cohort, 18% of the participants had pre-vaccination exposure to SARS-CoV-2 and this group had significantly higher anti-S titres post-second vaccine dose. In addition, 21% had evidence of exposure between the second and third vaccine dose, which may have contributed to further enhancing immune responses.

A central aim of the study was to determine the potential utility of measuring SARS-CoV-2 neutralising activity in the cohort. We first confirmed the reliability of cPass for detecting neutralisation of SARS-CoV-2 compared with PRNT using samples from vaccine recipients with HIV. We then found that detection of anti-S antibodies was largely concordant with the detection of neutralising responses by cPass, suggesting that anti-S antibodies can serve as a reliable proxy for neutralising capability in people with HIV. Notably however, a small subset of samples (12/144, 8.3%) showed anti-S antibodies in the absence of neutralising activity by cPass, with the proportion reduced to 8/144 (5.6%) if applying the manufacturer’s suggested anti-S titre threshold of 15 U/mL^32^. There were 3 samples that lacked neutralising activity by cPass despite anti-S titres ≥ 3.8 log_10_ U/mL, including 2 samples collected post-third dose.

In total, three participants lacked neutralising responses by cPass after three vaccine doses. Factors that might have contributed to the lack of neutralising capability include age ≥ 65^42^ and/or very low nadir and current CD4 count and CD4:CD8 ratio^42,43^. Interestingly however, the three individuals had detectable SARS-CoV-2 specific IFNγ responses. The IGRA approach used to measure T-cell reactivity to SARS-CoV-2 was previously validated against the ELISPOT assay^50^. As shown in recipients of immune-modifying therapies^51^ and illustrated here, IGRA and antibody test results may not agree completely, suggesting the potential utility of using IGRA to complement the assessment of immunity to SARS-CoV-2 in people with HIV. Despite defective humoral responses, T-cell responses may be detected that might still provide protection against COVID-19.

This study has several strengths, including a well-defined study population, a comprehensive methodology that aimed to evaluate practical immune assays including the novel SARS-CoV-2 IGRA, important confirmation that HIV infection does not modify the safety and tolerability profile of the BNT162b2 vaccine, interesting new observations on the effects of ethnicity and low-level viremia on humoral responses to SARS-CoV-2, and relevance to the formulation of clinical guidance. There are also several limitations related to size, duration of follow-up and correlation with clinical outcomes. In this small cohort, we did not find that anti-S and neutralising responses post-second vaccine dose influenced the likelihood of a subsequent SARS-CoV-2 infection, but numbers were small and larger studies with longer follow-up are needed to establish such correlation. More data are also needed to strengthen the preliminary observations regarding the potential utility of the SARS-CoV-2 IGRA, and a more complete data collection will be required to dissect the potential impact of comorbidities on vaccine immunogenicity. While studies are ongoing to explore the factors associated with reduced uptake of booster vaccination in the cohort, the findings have prompted the pro-active offer the third vaccine dose in the HIV clinic.

In conclusion, our study emphasises the importance of strengthening the offer of SARS-CoV-2 vaccination and boosting in people with HIV, particularly those who are older, show persistent immune dysfunction or viremia (even at low levels), or have a history of IDU. Vaccination is well tolerated in this population. Anti-S antibodies provide a reliable proxy for the presence of neutralisation activity, overall limiting the utility of surrogate virus neutralisation tests, whereas IGRA may provide a useful method for assessing T-cell immunity to SARS-CoV-2.

### Supplementary Information

Supplementary Information.

## Data Availability

The datasets generated and analysed during the current study are available from the corresponding author on reasonable request.
