# Supplementary material for: Utility of accessible SARS-CoV-2 specific immunoassays in vaccinated adults with a history of advanced HIV infection
Source: Sci Rep. 2024 Apr 9;14:8337. doi: 10.1038/s41598-024-58597-4 (PMC11003986; doi:10.1038/s41598-024-58597-4)

Utility of accessible SARS-CoV-2 specific immunoassays in vaccinated adults with a history of advanced HIV Infection

Ludovica Ferrari[1,2§], Alessandra Ruggiero[3§], Chiara Stefani[3], Livia Benedetti[1], Lorenzo Piermatteo[4], Eleonora Andreassi[5], Federica Caldara[2], Drieda Zace[2], Matteo Pagliari[6], Francesca Ceccherini Silberstein[5], Christopher Jones[7], Marco Iannetta[1,2], Anna Maria Geretti[2,8,9*] on behalf of the EVAN-COV Study Group

[§]Equal contribution

1. Dept. of Systems Medicine, University of Rome Tor Vergata, Rome, Italy
2. Dept. of Infectious Diseases, Fondazione PTV, University of Rome Tor Vergata, Rome, Italy
3. Dept. of Neurosciences, Biomedicine and Movement Sciences, School of Medicine, University of Verona, Verona, Italy
4. Department of Biology, University of Rome Tor Vergata, Rome, Italy
5. Dept. of Experimental Medicine, University of Rome Tor Vergata, Rome, Italy
6. Laboratory of Experimental Animal Models, Division of Comparative Biomedical Sciences, Istituto Zooprofilattico Sperimentale delle Venezie, Legnaro, Italy
7. Dept of Primary Care and Public Health, Brighton and Sussex Medical School, Falmer, UK
8. Dept. of Infection, North Middlesex University Hospital, London, UK
9. School of Immunity & Microbial Sciences, King's College London, London, UK

***Address correspondence to:**

Prof Anna Maria Geretti,

Dept. of Infectious Diseases, Fondazione PTV,

University of Rome Tor Vergata, Viale Oxford 81, 00133 Rome, Italy

Email: anna_maria.geretti@kcl.ac.uk

https://orcid.org/0000-0002-3670-6588

Supplementary File

Figure S1. Proportions reporting solicited local and systemic symptoms in the 7 days after each vaccine dose within the study population (PTV) and the total population of the pivotal BNT162b2 trials (31). P-values relate to the comparison between the study population and the trial population at each vaccine dose (*<0.05; ** <0.001; ***<0.0001).

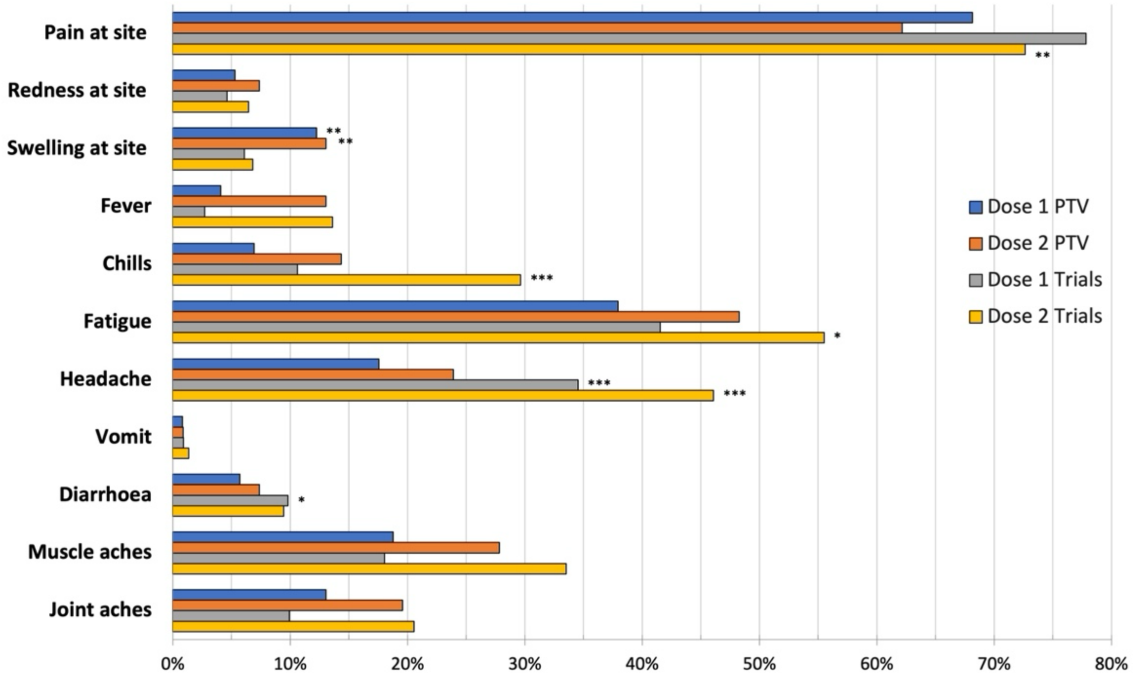

Table S1. Solicited and unsolicited side effects in the 7 days after each vaccine dose

		Dose 1 (n=245)		Dose 2 (n=230)	
**Participants with solicited symptoms**		N	%	N	%
≥1 symptom		191	78.0	175	76.1
≥1 local symptom		172	70.2	145	63.0
≥1 local severe symptom		9	3.7	11	4.8
≥1 systemic symptom		122	49.8	138	60.0
≥1 severe systemic symptom		13	5.3	26	11.3
**Participants with unsolicited symptoms**					
≥1 symptom		18	7.3	21	9.1
≥1 severe symptom		1	0.4	3	1.3
**Participants with solicited and/or unsolicited symptoms**					
≥1 symptom		192	78.4	175	76.1
≥1 severe symptom		20	8.2	32	13.9
**Type of solicited symptoms**					
Local	Pain	167	68.2	143	62.2
	Redness	13	5.3	17	7.4
	Swelling	30	12.2	30	13.0
Systemic	Fever	10	4.1	30	13.0
	Chills	17	6.9	33	14.3
	Fatigue	93	38.0	111	48.3
	Headache	43	17.6	55	23.9
	Vomiting	2	0.8	2	0.9
	Diarrhoea	14	5.7	17	7.4
	Muscle pain	46	18.8	64	27.8
	Joint pain	32	13.1	45	19.6
**Type of unsolicited symptoms**					
	Light-headiness, dizziness	4	1.6	4	1.7
	Sleep disturbances	3	1.2	2	0.9
	Loss of appetite, nausea	4	1.6	5	2.2
	Alteration of taste, dry mouth, gingivitis	2	0.8	3	1.3
	Upper respiratory tract symptoms	2	0.8	1	0.4
	Skin rash, itching	0	0.0	3	1.3
	Tachycardia, hot flushes	2	0.8	3	1.3
	Paraesthesia	1	0.4	1	0.4
	Thrombophlebitis	1	0.4	0	0.0
	Dysuria	0	0.0	1	0.4
	Exacerbation of pre-existing condition[b]	2	0.8	2	0.9

[a]Local indicates the vaccine injection site (upper arm). [b]Herpes labialis (n=2), toothache (n=2).

Figure S2. Neutralisation activity against wild-type SARS-CoV-2 (WT) and the Omicron BA.2 variant in samples collected from 28 people with HIV who received three doses of the BNT162b2 vaccine, measured by cPass.

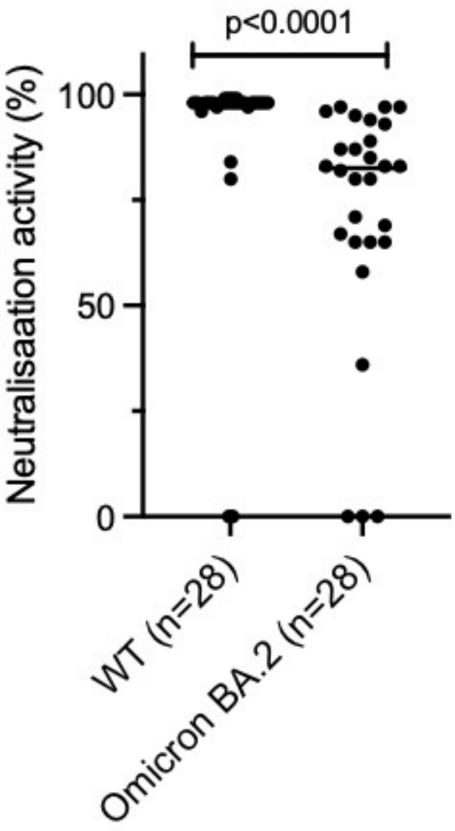

Supplement: Supplementary file 1 — Supplementary Information. [file 41598_2024_58597_MOESM1_ESM.pdf]
